# Dynamics Analysis of a Nonlinear Stochastic SEIR Epidemic System with Varying Population Size

**DOI:** 10.3390/e20050376

**Published:** 2018-05-17

**Authors:** Xiaofeng Han, Fei Li, Xinzhu Meng

**Affiliations:** 1 College of Mathematics and Systems Science, Shandong University of Science and Technology, Qingdao 266590, China; 2 State Key Laboratory of Mining Disaster Prevention and Control Co-founded by Shandong Province and the Ministry of Science and Technology, Shandong University of Science and Technology, Qingdao 266590, China

**Keywords:** Stochastic SEIR model, varying population size, vaccination, permanence in mean, stationary distribution

## Abstract

This paper considers a stochastic susceptible exposed infectious recovered (SEIR) epidemic model with varying population size and vaccination. We aim to study the global dynamics of the reduced nonlinear stochastic proportional differential system. We first investigate the existence and uniqueness of global positive solution of the stochastic system. Then the sufficient conditions for the extinction and permanence in mean of the infectious disease are obtained. Furthermore, we prove that the solution of the stochastic system has a unique ergodic stationary distribution under appropriate conditions. Finally, the discussion and numerical simulation are given to demonstrate the obtained results.

## 1. Introduction

Since the pioneering work of Kermack and Mckendrick [1], mathematical modeling for the dynamics of epidemic transmission has a realistic significance in predicting and controlling the spread of infectious diseases in the field of epidemiological research [2,3,4,5,6,7,8]. Recently, stochastic differential equations have been widely applied to physics, engineering, chemistry, and biology [9,10,11,12,13,14,15,16,17,18,19,20,21], which have obtained some novel results.

In fact, with the development of modern medicine, vaccination has become an important strategy for disease control. Then numerous scholars have investigated the effect of vaccination on disease [22,23,24,25,26,27]. The epidemic model with a constant population size is relatively effective for diseases with a low mortality and short duration. However, it is clearly untenable for diseases with a high mortality and varying populations. Thus epidemic models with varying population size seem to be more reasonable, which have attracted much interest from the research scientists [28,29,30]. Moreover, many infectious diseases incubate inside the hosts for a period of time before becoming infectious, so it is very meaningful to consider the effect of the incubation period. Based on the above considerations, Sun et al. [28] studied an SEIR model with varying population size and vaccination. The system can be described by

(1) $$\left\{\begin{array}{cc} \dot{S}= & bN-\frac{(1-\delta )\beta SI}{N}-\frac{\delta (1-p)\beta SI}{N}-\delta pS-\mu S,\\ \dot{E}= & \frac{(1-\delta )\beta SI}{N}+\frac{\delta (1-p)\beta SI}{N}-\alpha E-\mu E,\\ \dot{I}= & \alpha E-(\varepsilon +\gamma +\mu )I,\\ \dot{R}= & \delta pS+\gamma I-\mu R, \end{array}\right.$$

where S(t), E(t), I(t) and R(t), respectively, stand for the densities of the susceptible, the exposed, the infective and recovered individuals at time *t*, the total population size is denoted by N(t)=S(t)+E(t)+I(t)+R(t). *b* represents the inflow rate (including birth and immigration), μ denotes the outflow rate (including natural death and emigration). The function $\frac{\beta SI}{N}$ stands for the standard incidence rate, here β represents the transmission rate of disease. δ(0≤δ<1) is the vaccine coverage rate of susceptible individuals, *p*(0≤p≤1) is the vaccine efficacy, α represents the rate at which the exposed individuals become infectious, ε is the rate of disease-related death and γ stands for the recovery rate of infective individuals. The parameters δ and *p* are all non-negative constants and *b*, μ, β, α, ε and γ are positive constants. Moreover, the differential equation of total population size N(t) is given by $\dot{N}=\left(b-\mu \right)N-\varepsilon I$. The authors [28] explored the proportions of individuals in the four epidemiological classes, namely

(2) $$\tilde{s}=\frac{S}{N},\;\tilde{e}=\frac{E}{N},\;\tilde{i}=\frac{I}{N},\;\tilde{r}=\frac{R}{N}.$$

It is easy to get that the variables $\tilde{s}$, $\tilde{e}$, $\tilde{i}$ and $\tilde{r}$ satisfy the following system of differential equations

$$\left\{\begin{array}{cc} \dot{\tilde{s}}= & b-\left(1-\delta p\right)\beta \tilde{s}\tilde{i}-\left(\delta p+b\right)\tilde{s}+\varepsilon \tilde{s}\tilde{i},\\ \dot{\tilde{e}}= & \left(1-\delta p\right)\beta \tilde{s}\tilde{i}-\left(\alpha +b\right)\tilde{e}+\varepsilon \tilde{e}\tilde{i},\\ \dot{\tilde{i}}= & \alpha \tilde{e}-\left(\varepsilon +\gamma +b\right)\tilde{i}+\varepsilon {\tilde{i}}^{2},\\ \dot{\tilde{r}}= & \delta p\tilde{s}+\gamma \tilde{i}-b\tilde{r}+\varepsilon \tilde{i}\tilde{r}. \end{array}\right.$$

Since variable $\tilde{r}$ does not appear in the first, second, third equations of the above system. Then the above system becomes the following reduced system

(3) $$\left\{\begin{array}{cc} \dot{\tilde{s}}= & b-\left(1-\delta p\right)\beta \tilde{s}\tilde{i}-\left(\delta p+b\right)\tilde{s}+\varepsilon \tilde{s}\tilde{i},\\ \dot{\tilde{e}}= & \left(1-\delta p\right)\beta \tilde{s}\tilde{i}-\left(\alpha +b\right)\tilde{e}+\varepsilon \tilde{e}\tilde{i},\\ \dot{\tilde{i}}= & \alpha \tilde{e}-\left(\varepsilon +\gamma +b\right)\tilde{i}+\varepsilon {\tilde{i}}^{2} \end{array}\right.$$

which is subject to the constraint $\tilde{r}=1-\tilde{s}-\tilde{e}-\tilde{i}$. In the region $△=\left\{\left(\tilde{s},\tilde{e},\tilde{i}\right)\in R_{+}^{3}|0\leq \tilde{s}+\tilde{e}+\tilde{i}\leq 1\right\}$, they established the epidemiological threshold condition $R_{0}$, which determines disease extinction or permanence, where

$$R_{0}=\frac{b\alpha \beta (1-\delta p)}{\left(\alpha +b)(\delta p+b)(\varepsilon +\gamma +b\right)}.$$

Meanwhile, they analyzed the global dynamics of system (3) and derived the equilibria (including the disease-free equilibrium and the endemic equilibrium) and their global stability. In addition, the parameter restrictions for uniform permanence were obtained.

Nevertheless, the biological populations in the ecosystem are inevitably subjected to uncertain environmental perturbations. It is worth noting that this phenomenon is ubiquitous in the natural environment. So various stochastic epidemic models have been proposed and studied [31,32,33,34,35,36]. To the best of our knowledge, there are not too many researches on global dynamics of the stochastic SEIR epidemic model with varying population size and vaccination yet. In this paper, to make this epidemic model (1) more reasonable and realistic, we suppose the stochastic perturbations are directly proportional to $\tilde{s}$, $\tilde{e}$, $\tilde{i}$ and $\tilde{r}$ under the influence of white noise type, influenced on the $\dot{\tilde{s}}\left(t\right)$, $\dot{\tilde{e}}\left(t\right)$, $\dot{\tilde{i}}\left(t\right)$ and $\dot{\tilde{r}}\left(t\right)$ in system (1), respectively. This implies the stochastic effects of white noise on the birth and death rates of S,E,I,R. Then corresponding to system (1), a stochastic version can be reached by

(4) $$\left\{\begin{array}{cc} dS= & \left[bN-\frac{(1-\delta )\beta SI}{N}-\frac{\delta (1-p)\beta SI}{N}-\delta pS-\mu S\right]dt+\sigma_{1}SdB_{1}\left(t\right),\\ dE= & \left[\frac{(1-\delta )\beta SI}{N}+\frac{\delta (1-p)\beta SI}{N}-\alpha E-\mu E\right]dt+\sigma_{2}EdB_{2}\left(t\right),\\ dI= & \left[\alpha E-(\varepsilon +\gamma +\mu )I\right]dt+\sigma_{3}IdB_{3}\left(t\right),\\ dR= & \left(\delta pS+\gamma I-\mu R\right)dt+\sigma_{4}RdB_{4}\left(t\right), \end{array}\right.$$

where $B_{i}\left(t\right)\left(i=1,2,3,4\right)$ is the standard Wiener processes with $B_{i}\left(0\right)=0$ a.s. $\sigma_{i}\left(t\right)\left(i=1,2,3,4\right)$ stands for a continuous and bounded function for any t≥0 and $\sigma_{i}^{2}\left(t\right)\left(i=1,2,3,4\right)$ represents the intensities of Wiener processes. Furthermore, the differential equation of total population size N(t) is given by the following form

$$dN=\left[\left(b-\mu \right)N-\varepsilon I\right]dt+\sigma_{1}SdB_{1}\left(t\right)+\sigma_{2}EdB_{2}\left(t\right)+\sigma_{3}IdB_{3}\left(t\right)+\sigma_{4}RdB_{4}\left(t\right).$$

From (2), the system (4) becomes the following proportional system

(5) $$\left\{\begin{array}{cc} d\tilde{s}= & \left[b-\left(1-\delta p\right)\beta \tilde{s}\tilde{i}-\left(\delta p+b\right)\tilde{s}+\varepsilon \tilde{s}\tilde{i}-\sigma_{1}^{2}{\tilde{s}}^{2}+\tilde{s}\left(\sigma_{1}^{2}{\tilde{s}}^{2}+\sigma_{2}^{2}{\tilde{e}}^{2}+\sigma_{3}^{2}{\tilde{i}}^{2}+\sigma_{4}^{2}{\tilde{r}}^{2}\right)\right]dt\\ & +\sigma_{1}\tilde{s}\left(1-\tilde{s}\right)dB_{1}\left(t\right)-\sigma_{2}\tilde{s}\tilde{e}dB_{2}\left(t\right)-\sigma_{3}\tilde{s}\tilde{i}dB_{3}\left(t\right)-\sigma_{4}\tilde{s}\tilde{r}dB_{4}\left(t\right),\\ d\tilde{e}= & \left[\left(1-\delta p\right)\beta \tilde{s}\tilde{i}-\left(\alpha +b\right)\tilde{e}+\varepsilon \tilde{e}\tilde{i}-\sigma_{2}^{2}{\tilde{e}}^{2}+\tilde{e}\left(\sigma_{1}^{2}{\tilde{s}}^{2}+\sigma_{2}^{2}{\tilde{e}}^{2}+\sigma_{3}^{2}{\tilde{i}}^{2}+\sigma_{4}^{2}{\tilde{r}}^{2}\right)\right]dt\\ & -\sigma_{1}\tilde{s}\tilde{e}dB_{1}\left(t\right)+\sigma_{2}\tilde{e}\left(1-\tilde{e}\right)dB_{2}\left(t\right)-\sigma_{3}\tilde{e}\tilde{i}dB_{3}\left(t\right)-\sigma_{4}\tilde{e}\tilde{r}dB_{4}\left(t\right),\\ d\tilde{i}= & \left[\alpha \tilde{e}-\left(\varepsilon +\gamma +b\right)\tilde{i}+\varepsilon {\tilde{i}}^{2}-\sigma_{3}^{2}{\tilde{i}}^{2}+\tilde{i}\left(\sigma_{1}^{2}{\tilde{s}}^{2}+\sigma_{2}^{2}{\tilde{e}}^{2}+\sigma_{3}^{2}{\tilde{i}}^{2}+\sigma_{4}^{2}{\tilde{r}}^{2}\right)\right]dt\\ & -\sigma_{1}\tilde{s}\tilde{i}dB_{1}\left(t\right)-\sigma_{2}\tilde{e}\tilde{i}dB_{2}\left(t\right)+\sigma_{3}\tilde{i}\left(1-\tilde{i}\right)dB_{3}\left(t\right)-\sigma_{4}\tilde{i}\tilde{r}dB_{4}\left(t\right),\\ d\tilde{r}= & \left[\delta p\tilde{s}+\gamma \tilde{i}-b\tilde{r}+\varepsilon \tilde{i}\tilde{r}-\sigma_{4}^{2}{\tilde{r}}^{2}+\tilde{r}\left(\sigma_{1}^{2}{\tilde{s}}^{2}+\sigma_{2}^{2}{\tilde{e}}^{2}+\sigma_{3}^{2}{\tilde{i}}^{2}+\sigma_{4}^{2}{\tilde{r}}^{2}\right)\right]dt\\ & -\sigma_{1}\tilde{s}\tilde{r}dB_{1}\left(t\right)-\sigma_{2}\tilde{e}\tilde{r}dB_{2}\left(t\right)-\sigma_{3}\tilde{i}\tilde{r}dB_{3}\left(t\right)+\sigma_{4}\tilde{r}\left(1-\tilde{r}\right)dB_{4}\left(t\right). \end{array}\right.$$

It is worthy to note that, the variables $\tilde{s}$, $\tilde{e}$, $\tilde{i}$ and $\tilde{r}$ satisfy the relation $\tilde{r}=1-\tilde{s}-\tilde{e}-\tilde{i}$, we can omit analysis of the fourth equation of system (5) and explore the following reduced system

(6) $$\left\{\begin{array}{cc} d\tilde{s}= & [b-\left(1-\delta p\right)\beta \tilde{s}\tilde{i}-\left(\delta p+b\right)\tilde{s}+\varepsilon \tilde{s}\tilde{i}-\sigma_{1}^{2}{\tilde{s}}^{2}+\tilde{s}(\sigma_{1}^{2}{\tilde{s}}^{2}+\sigma_{2}^{2}{\tilde{e}}^{2}+\sigma_{3}^{2}{\tilde{i}}^{2}\\ & +\sigma_{4}^{2}{\left(1-\tilde{s}-\tilde{e}-\tilde{i}\right)}^{2})]dt+\sigma_{1}\tilde{s}\left(1-\tilde{s}\right)dB_{1}\left(t\right)-\sigma_{2}\tilde{s}\tilde{e}dB_{2}\left(t\right)-\sigma_{3}\tilde{s}\tilde{i}dB_{3}\left(t\right)\\ & -\sigma_{4}\tilde{s}\left(1-\tilde{s}-\tilde{e}-\tilde{i}\right)dB_{4}\left(t\right),\\ d\tilde{e}= & [\left(1-\delta p\right)\beta \tilde{s}\tilde{i}-\left(\alpha +b\right)\tilde{e}+\varepsilon \tilde{e}\tilde{i}-\sigma_{2}^{2}{\tilde{e}}^{2}+\tilde{e}(\sigma_{1}^{2}{\tilde{s}}^{2}+\sigma_{2}^{2}{\tilde{e}}^{2}+\sigma_{3}^{2}{\tilde{i}}^{2}\\ & +\sigma_{4}^{2}{\left(1-\tilde{s}-\tilde{e}-\tilde{i}\right)}^{2})]dt-\sigma_{1}\tilde{s}\tilde{e}dB_{1}\left(t\right)+\sigma_{2}\tilde{e}\left(1-\tilde{e}\right)dB_{2}\left(t\right)-\sigma_{3}\tilde{e}\tilde{i}dB_{3}\left(t\right)\\ & -\sigma_{4}\tilde{e}\left(1-\tilde{s}-\tilde{e}-\tilde{i}\right)dB_{4}\left(t\right),\\ d\tilde{i}= & \left[\alpha \tilde{e}-\left(\varepsilon +\gamma +b\right)\tilde{i}+\varepsilon {\tilde{i}}^{2}-\sigma_{3}^{2}{\tilde{i}}^{2}+\tilde{i}\left(\sigma_{1}^{2}{\tilde{s}}^{2}+\sigma_{2}^{2}{\tilde{e}}^{2}+\sigma_{3}^{2}{\tilde{i}}^{2}+\sigma_{4}^{2}{\left(1-\tilde{s}-\tilde{e}-\tilde{i}\right)}^{2}\right)\right]dt\\ & -\sigma_{1}\tilde{s}\tilde{i}dB_{1}\left(t\right)-\sigma_{2}\tilde{e}\tilde{i}dB_{2}\left(t\right)+\sigma_{3}\tilde{i}\left(1-\tilde{i}\right)dB_{3}\left(t\right)-\sigma_{4}\tilde{i}\left(1-\tilde{s}-\tilde{e}-\tilde{i}\right)dB_{4}\left(t\right) \end{array}\right.$$

with the initial value $\left(\tilde{s}\left(0\right),\tilde{e}\left(0\right),\tilde{i}\left(0\right)\right)\in R_{+}^{3}$ and $\tilde{s}\left(0\right)+\tilde{e}\left(0\right)+\tilde{i}\left(0\right)<1$.

Since system (6) is a three-dimensional stochastic system with many high-order nonlinear terms, this makes the stochastic analysis novel and more complex than [34,36].

Throughout this article, unless otherwise specified, let $\left(\Omega ,F,{\left\{F\right\}}_{t\geq 0},P\right)$ be a complete probability space with a filtration ${\left\{F_{t}\right\}}_{t\geq 0}$ satisfying the usual conditions (i.e., it is increasing and right continuous while $F_{0}$ contains all P-null sets). Further suppose $B_{i}\left(t\right)\left(i=1,2,3,4\right)$ stands for the mutually independent standard Wiener processes defined on the complete probability space Ω. For an integrable function x(t) on [0,+∞), let us define $\langle x\left(t\right)\rangle =\frac{1}{t}\int_{0}^{t}x\left(r\right)dr$.

## 2. Global Positive Solution

The following Itô’s formula will be used frequently in the sequel.

**Lemma** **1.** *[37] Assume that $X\left(t\right)\in R^{+}$ is an Itô’s process of the form*

$$dX\left(t\right)=F\left(X\left(t^{-}\right),t^{-}\right)dt+G\left(X\left(t^{-}\right),t^{-}\right)dB\left(t\right),$$

*where $F:R^{n}\times R_{+}\times S\to R^{n}$ and $\;G:R^{n}\times R_{+}\times S\to R^{n}$ are measurable functions.*

*Given $V\in C^{2,1}\left(R^{n}\times R_{+}\times S;R_{+}\right)$, we define the operator LV by*

$$\begin{array}{cc} LV(X,t)= & V_{t}\left(X,t\right)+V_{X}\left(X,t\right)F\left(X,t\right)+\frac{1}{2}\mathrm{trace}\left[G^{T}\left(X,t\right)V_{XX}\left(X,t\right)G\left(X,t\right)\right], \end{array}$$

*where*

$$V_{t}\left(X,t\right)=\frac{\partial V_{X}\left(X,t\right)}{\partial t},\;V_{X}\left(X,t\right)=\left(\frac{\partial V_{X}\left(X,t\right)}{\partial X_{1}},\cdots ,\frac{\partial V_{X}\left(X,t\right)}{\partial X_{n}}\right),\;V_{XX}\left(X,t\right)={\left(\frac{\partial^{2}V_{X}\left(X,t\right)}{\partial X_{i}\partial X_{j}}\right)}_{n\times n}.$$

*Then the generalized Itô’s formula is given by*

$$\begin{array}{cc} dV(X,t)= & LV\left(X,t\right)dt+V_{X}\left(X,t\right)G\left(X,t\right)dB\left(t\right). \end{array}$$

To explore the dynamical behaviors of a population system, we first concern the global existence and positivity of the solutions of system (6).

**Lemma** **2.** *For any given initial value $\left(\tilde{s}\left(0\right),\tilde{e}\left(0\right),\tilde{i}\left(0\right)\right)\in R_{+}^{3}$ and $\tilde{s}\left(0\right)+\tilde{e}\left(0\right)+\tilde{i}\left(0\right)<1$, the system (6) has a unique positive local solution $\left(\tilde{s}\left(t\right),\tilde{e}\left(t\right),\tilde{i}\left(t\right)\right)$ for $t\in [-\omega ,\tau_{e})$, where $\tau_{e}$ is the explosion time [37].*

**Theorem** **1.** *For any given initial value $\left(\tilde{s}\left(0\right),\tilde{e}\left(0\right),\tilde{i}\left(0\right)\right)\in R_{+}^{3}$ and $\tilde{s}\left(0\right)+\tilde{e}\left(0\right)+\tilde{i}\left(0\right)<1$, the system (6) has a unique positive solution $\left(\tilde{s}\left(t\right),\tilde{e}\left(t\right),\tilde{i}\left(t\right)\right)\in R_{+}^{3}$ on t>0 a.s.*

**Proof.**  The following proof is divided into two parts.

**Part I.** Since the coefficients of the system (6) satisfy local Lipschitz condition, from Lemma 2, it is easy to see that the system (6) has a unique positive local solution $\left(\tilde{s}\left(t\right),\tilde{e}\left(t\right),\tilde{i}\left(t\right)\right)$ for any given initial value $\left(\tilde{s}\left(0\right),\tilde{e}\left(0\right),\tilde{i}\left(0\right)\right)\in R_{+}^{3}$ and $\tilde{s}\left(0\right)+\tilde{e}\left(0\right)+\tilde{i}\left(0\right)<1$.

**Part II.** Now we prove that the positive solution is global, that is $\tau_{e}=\infty$ a.s. Let $k_{0}\geq 0$ be sufficiently large such that $\tilde{s}\left(0\right)$, $\tilde{e}\left(0\right)$ and $\tilde{i}\left(0\right)$ all lie in $\left[\frac{1}{k_{0}},k_{0}\right]$. For each integer $k\geq k_{0}$, let us define the stopping time

$$\tau_{k}=\inf\left\{t\in \left[-\omega ,\tau_{e}\right):\tilde{s}\left(t\right)\notin \left(\frac{1}{k},k\right),\;\tilde{e}\left(t\right)\notin \left(\frac{1}{k},k\right)\;\mathrm{or}\;\tilde{i}\left(t\right)\notin \left(\frac{1}{k},k\right)\right\},$$

where we define inf∅=∞ (∅ stands for the empty set). Evidently, $\tau_{k}$ is strictly increasing when k→∞. Let $\tau_{\infty }=\lim_{k\to \infty }\tau_{k}$, thus $\tau_{\infty }\leq \tau_{e}$ a.s. So we just need to show that $\tau_{\infty }=\infty$ a.s. If $\tau_{\infty }=\infty$ is untrue, then there exist two constants T>0 and ϵ∈(0,1) such that $P\{\tau_{\infty }\leq T\}>\epsilon$. Hence, there exists $k_{1}\geq k_{0}\left(k_{1}\in N_{+}\right)$ such that

(7) $$\begin{array}{c} P\left\{\tau_{k}\leq T\right\}\geq \epsilon ,\;k\geq k_{1}. \end{array}$$

Define a $C^{2}$-function *V*: $R_{+}^{3}\to R_{+}$ by

$$\begin{array}{cc} V\left(\tilde{s},\tilde{e},\tilde{i}\right)= & -\ln\left(1-\tilde{s}-\tilde{e}-\tilde{i}\right)-\ln\tilde{s}-\ln\tilde{e}-\ln\tilde{i}-3. \end{array}$$

The non-negativity of $V(\tilde{s},\tilde{e},\tilde{i})$ can be obtained from m−1−lnm≥0,m>0.

In terms of the multi-dimensional Itô’s formula and system (6), we have

$$\begin{array}{cc} dV= & LVdt+\sigma_{1}\left(4\tilde{s}-1\right)dB_{1}\left(t\right)+\sigma_{2}\left(4\tilde{e}-1\right)dB_{2}\left(t\right)+\sigma_{3}\left(4\tilde{i}-1\right)dB_{3}\left(t\right)\\ & +\sigma_{4}\left(3-4\tilde{s}-4\tilde{e}-4\tilde{i}\right)dB_{4}\left(t\right), \end{array}$$

where LV is given in Appendix A in detail. Then we have

$$\begin{array}{cc} LV\leq & \beta +\delta p+4b+\varepsilon +\alpha +\gamma +\frac{1}{2}\sigma_{1}^{2}+\frac{1}{2}\sigma_{2}^{2}+\frac{1}{2}\sigma_{3}^{2}+\frac{1}{2}\sigma_{4}^{2}\\ := & M_{0}, \end{array}$$

where $M_{0}$ is a positive constant.

So we get

(8) $$\begin{array}{cc} dV\leq & M_{0}dt+\sigma_{1}\left(4\tilde{s}-1\right)dB_{1}\left(t\right)+\sigma_{2}\left(4\tilde{e}-1\right)dB_{2}\left(t\right)+\sigma_{3}\left(4\tilde{i}-1\right)dB_{3}\left(t\right)\\ & +\sigma_{4}\left(3-4\tilde{s}-4\tilde{e}-4\tilde{i}\right)dB_{4}\left(t\right). \end{array}$$

Integrating both sides of (8) from 0 to $\tau_{k}\wedge T$ and then taking the expectation yield

(9) $$\begin{array}{cc} EV\left(\tilde{s}\left(\tau_{k}\wedge T\right),\tilde{e}\left(\tau_{k}\wedge T\right),\tilde{i}\left(\tau_{k}\wedge T\right)\right)\leq & V\left(\tilde{s}\left(0\right),\tilde{e}\left(0\right),\tilde{i}\left(0\right)\right)+E\int_{0}^{\tau_{k}\wedge T}M_{0}dt\\ \leq & V\left(\tilde{s}\left(0\right),\tilde{e}\left(0\right),\tilde{i}\left(0\right)\right)+M_{0}T. \end{array}$$

Let $\Omega_{k}=\left\{\tau_{k}\leq T\right\},\;k\geq k_{1}$ and from (7), we have $P(\Omega_{k})\geq \epsilon$. Notice that for every $\omega \in \Omega_{k}$, there exists $\tilde{s}\left(\tau_{k},\omega \right),\tilde{e}\left(\tau_{k},\omega \right)$ or $\tilde{i}\left(\tau_{k},\omega \right)$ equals either $\frac{1}{k}$ or *k*. Thus

(10) $$\begin{array}{c} V\left(\tilde{s}\left(\tau_{k},\omega \right),\tilde{e}\left(\tau_{k},\omega \right),\tilde{i}\left(\tau_{k},\omega \right)\right)\geq \left(\frac{1}{k}-1-\ln\frac{1}{k}\right)\wedge \left(k-1-\ln k\right). \end{array}$$

By virtue of (9) and (10), we have

$$\begin{array}{cc} V\left(\tilde{s}\left(0\right),\tilde{e}\left(0\right),\tilde{i}\left(0\right)\right)+M_{0}T\geq & E\left[1_{\Omega_{k}\left(\omega \right)}V\left(\tilde{s}\left(\tau_{k},\omega \right),\tilde{e}\left(\tau_{k},\omega \right),\tilde{i}\left(\tau_{k},\omega \right)\right)\right]\\ \geq & \epsilon \left[\left(\frac{1}{k}-1-\ln\frac{1}{k}\right)\wedge \left(k-1-\ln k\right)\right], \end{array}$$

here $1_{\Omega_{k}\left(\omega \right)}$ represents the indicator function of $\Omega_{k}\left(\omega \right)$.

Let k→∞, which implies

$$\infty >V\left(\tilde{s}\left(0\right),\tilde{e}\left(0\right),\tilde{i}\left(0\right)\right)+M_{0}T=\infty$$

is a contradiction. Obviously, we get that $\tau_{\infty }=\infty$. The proof of Theorem 1 is complete.  ☐

## 3. Extinction

For a population system, the parameter conditions of disease extinction and permanence have become an important issue that attracts more and more attention in real life. In this section, we mainly investigate the extinction of disease and leave the argument of permanence to the next section.

**Theorem** **2.** *Let $\left(\tilde{s}\left(t\right),\tilde{e}\left(t\right),\tilde{i}\left(t\right)\right)$ be the solution of system (6) with the initial value $\left(\tilde{s}\left(0\right),\tilde{e}\left(0\right),\tilde{i}\left(0\right)\right)$$\in R_{+}^{3}$ and $\tilde{s}\left(0\right)+\tilde{e}\left(0\right)+\tilde{i}\left(0\right)<1$. If the parameter conditions*

$$M_{1}<2\left(1-\varrho \right)\alpha ,\;\varepsilon \leq \left(1-\delta p\right)\beta <\varepsilon +\gamma +b$$

*hold, then*

$$\underset{t\to \infty }{\lim\;\sup}\frac{\ln\left(\tilde{e}\left(t\right)+\varrho \tilde{i}\left(t\right)\right)}{t}\leq \left(\varrho -1\right)\alpha +\frac{M_{1}}{2}<0\;a.s.,$$

*where*

$$\varrho =\frac{-\left(\varepsilon +\gamma +b-\alpha \right)+\sqrt{{\left(\varepsilon +\gamma +b-\alpha \right)}^{2}+4\left(1-\delta p\right)\alpha \beta }}{2\alpha }$$

*and*

$$M_{1}=\max\left\{\sigma_{1}^{2},\left(1-\varrho^{2}\right)\sigma_{3}^{2},\sigma_{4}^{2}\right\},$$

*namely, $\tilde{e}\left(t\right)$ and $\tilde{i}\left(t\right)$ tend to zero exponentially a.s. That is to say, the exposed and infective individuals go to extinction almost surely.*

**Proof.**  Let us define a differentiable function *V* by

$$V=\ln\left(\tilde{e}\left(t\right)+\varrho \tilde{i}\left(t\right)\right),$$

here ϱ is a positive constant to be determined later. According to the Itô’s formula and system (6), we have

(11) $$\begin{array}{cc} dV= & LVdt-\sigma_{1}\tilde{s}dB_{1}\left(t\right)+\frac{\sigma_{2}\tilde{e}\left(1-\tilde{e}-\varrho \tilde{i}\right)}{\tilde{e}+\varrho \tilde{i}}dB_{2}\left(t\right)+\frac{\sigma_{3}\tilde{i}\left(\varrho -\tilde{e}-\varrho \tilde{i}\right)}{\tilde{e}+\varrho \tilde{i}}dB_{3}\left(t\right)\\ & -\sigma_{4}\left(1-\tilde{s}-\tilde{e}-\tilde{i}\right)dB_{4}\left(t\right), \end{array}$$

where LV is given in Appendix B in detail. One can derive that

(12) $$\begin{array}{cc} dV\leq & \left[\left(\varrho -1\right)\alpha +\frac{M_{1}}{2}\right]dt-\sigma_{1}\tilde{s}dB_{1}\left(t\right)+\frac{\sigma_{2}\tilde{e}\left(1-\tilde{e}-\varrho \tilde{i}\right)}{\tilde{e}+\varrho \tilde{i}}dB_{2}\left(t\right)+\frac{\sigma_{3}\tilde{i}\left(\varrho -\tilde{e}-\varrho \tilde{i}\right)}{\tilde{e}+\varrho \tilde{i}}dB_{3}\left(t\right)\\ & -\sigma_{4}\left(1-\tilde{s}-\tilde{e}-\tilde{i}\right)dB_{4}\left(t\right), \end{array}$$

here $M_{1}=\max\left\{\sigma_{1}^{2},\left(1-\varrho^{2}\right)\sigma_{3}^{2},\sigma_{4}^{2}\right\}$. Then, integrating from 0 to *t* and dividing by *t* on both sides of (12) yield

$$\begin{array}{c} \frac{\ln\left(\tilde{e}\left(t\right)+\varrho \tilde{i}\left(t\right)\right)}{t}\leq \left(\varrho -1\right)\alpha +\frac{M_{1}}{2}+\frac{\ln\left(\tilde{e}\left(0\right)+\varrho \tilde{i}\left(0\right)\right)}{t}+\frac{\tilde{M}\left(t\right)}{t}, \end{array}$$

here

$$\begin{array}{cc} \tilde{M}\left(t\right)= & -\sigma_{1}\int_{0}^{t}\tilde{s}\left(r\right)dB_{1}\left(r\right)+\sigma_{2}\int_{0}^{t}\frac{\tilde{e}\left(r\right)\left(1-\tilde{e}\left(r\right)-\varrho \tilde{i}\left(r\right)\right)}{\tilde{e}\left(r\right)+\varrho \tilde{i}\left(r\right)}dB_{2}\left(r\right)\\ & +\sigma_{3}\int_{0}^{t}\frac{\tilde{i}\left(r\right)\left(\varrho -\tilde{e}\left(r\right)-\varrho \tilde{i}\left(r\right)\right)}{\tilde{e}\left(r\right)+\varrho \tilde{i}\left(r\right)}dB_{3}\left(r\right)-\sigma_{4}\int_{0}^{t}\left(1-\tilde{s}\left(r\right)-\tilde{e}\left(r\right)-\tilde{i}\left(r\right)\right)dB_{4}\left(r\right). \end{array}$$

In a similar way as [38], making use of the strong law of large numbers [37] yields

$$\lim_{t\to \infty }\frac{\tilde{M}\left(t\right)}{t}=0\;a.s.$$

Therefore,

$$\underset{t\to \infty }{\lim\;\sup}\frac{\ln\left(\tilde{e}\left(t\right)+\varrho \tilde{i}\left(t\right)\right)}{t}\leq \left(\varrho -1\right)\alpha +\frac{M_{1}}{2}<0\;a.s.,$$

which shows that

$$\lim_{t\to \infty }\tilde{e}\left(t\right)=0,\;\lim_{t\to \infty }\tilde{i}\left(t\right)=0\;a.s.$$

The proof of Theorem 2 is complete. ☐

## 4. Permanence in Mean

**Theorem** **3.** *Let $\left(\tilde{s}\left(t\right),\tilde{e}\left(t\right),\tilde{i}\left(t\right)\right)$ be the solution of system (6) with the initial value $\left(\tilde{s}\left(0\right),\tilde{e}\left(0\right),\tilde{i}\left(0\right)\right)$$\in R_{+}^{3}$ and $\tilde{s}\left(0\right)+\tilde{e}\left(0\right)+\tilde{i}\left(0\right)<1$. If the parameter condition*

$$\sqrt[{3}]{b(1-\delta p)\beta \alpha }>\frac{\delta p+3b+\alpha +\varepsilon +\gamma +\frac{1}{2}\sigma_{1}^{2}+\frac{1}{2}\sigma_{2}^{2}+\frac{1}{2}\sigma_{3}^{2}}{3}$$

*holds, then*

$$\underline{\tilde{i}}\leq \underset{t\to \infty }{\lim\;\inf}\langle \tilde{i}\left(t\right)\rangle \leq \underset{t\to \infty }{\lim\;\sup}\langle \tilde{i}\left(t\right)\rangle \leq \bar{\tilde{i}}\;a.s.,$$

*where*

$$\underline{\tilde{i}}=\frac{3\sqrt[{3}]{b(1-\delta p)\beta \alpha }-\left(\delta p+3b+\alpha +\varepsilon +\gamma +\frac{1}{2}\sigma_{1}^{2}+\frac{1}{2}\sigma_{2}^{2}+\frac{1}{2}\sigma_{3}^{2}\right)}{(1-\delta p)\beta }$$

*and*

$$\bar{\tilde{i}}=\frac{2b+\alpha +\varepsilon +\gamma +\frac{1}{2}\sigma_{2}^{2}+\frac{1}{2}\sigma_{3}^{2}}{2\varepsilon },$$

*that is to say, the infective individuals $\tilde{i}\left(t\right)$ are permanent in mean almost surely.*

**Proof.**  The following proof is divided into two steps.

**Step I.** According to the Itô’s formula and system (6), we have

(13) $$\begin{array}{cc} d\left(\ln\tilde{s}+\ln\tilde{e}+\ln\tilde{i}\right)= & [\frac{b}{\tilde{s}}-\left(1-\delta p\right)\beta \tilde{i}+3\varepsilon \tilde{i}+\frac{\left(1-\delta p\right)\beta \tilde{s}\tilde{i}}{\tilde{e}}+\frac{\alpha \tilde{e}}{\tilde{i}}+\frac{3}{2}\sigma_{1}^{2}{\tilde{s}}^{2}+\frac{3}{2}\sigma_{2}^{2}{\tilde{e}}^{2}+\frac{3}{2}\sigma_{3}^{2}{\tilde{i}}^{2}\\ & +\frac{3}{2}\sigma_{4}^{2}{\left(1-\tilde{s}-\tilde{e}-\tilde{i}\right)}^{2}-(\delta p+3b+\alpha +\varepsilon +\gamma +\frac{1}{2}\sigma_{1}^{2}+\frac{1}{2}\sigma_{2}^{2}\\ & +\frac{1}{2}\sigma_{3}^{2})]dt+\sigma_{1}\left(1-3\tilde{s}\right)dB_{1}\left(t\right)+\sigma_{2}\left(1-3\tilde{e}\right)dB_{2}\left(t\right)\\ & +\sigma_{3}\left(1-3\tilde{i}\right)dB_{3}\left(t\right)-3\sigma_{4}\left(1-\tilde{s}-\tilde{e}-\tilde{i}\right)dB_{4}\left(t\right). \end{array}$$

Integrating from 0 to *t* and dividing by *t* on both sides of (13) lead to

$$\begin{array}{cc} \frac{\ln\tilde{s}\left(t\right)}{t}+\frac{\ln\tilde{e}\left(t\right)}{t}+\frac{\ln\tilde{i}\left(t\right)}{t}\geq & 3\sqrt[{3}]{b(1-\delta p)\beta \alpha }-\left(1-\delta p\right)\beta \langle \tilde{i}\rangle -(\delta p+3b+\alpha +\varepsilon +\gamma \\ & +\frac{1}{2}\sigma_{1}^{2}+\frac{1}{2}\sigma_{2}^{2}+\frac{1}{2}\sigma_{3}^{2})+\frac{\ln\tilde{s}\left(0\right)}{t}+\frac{\ln\tilde{e}\left(0\right)}{t}+\frac{\ln\tilde{i}\left(0\right)}{t}+\frac{\underline{M}\left(t\right)}{t}, \end{array}$$

here

$$\begin{array}{cc} \underline{M}\left(t\right)= & \sigma_{1}\int_{0}^{t}\left(1-3\tilde{s}\left(r\right)\right)dB_{1}\left(r\right)+\sigma_{2}\int_{0}^{t}\left(1-3\tilde{e}\left(r\right)\right)dB_{2}\left(r\right)+\sigma_{3}\int_{0}^{t}\left(1-3\tilde{i}\left(r\right)\right)dB_{3}\left(r\right)\\ & -3\sigma_{4}\int_{0}^{t}\left(1-\tilde{s}\left(r\right)-\tilde{e}\left(r\right)-\tilde{i}\left(r\right)\right)dB_{4}\left(r\right). \end{array}$$

The detail derivation process for the above inequality is given in Appendix C.

In a similar way as [38], making use of the strong law of large numbers [37] leads to

$$\lim_{t\to \infty }\frac{\underline{M}\left(t\right)}{t}=0\;a.s.$$

Then, by virtue of $-\infty <\ln\tilde{s}\left(t\right)<0$, $-\infty <\ln\tilde{e}\left(t\right)<0$, $-\infty <\ln\tilde{i}\left(t\right)<0$$\left(\tilde{s}+\tilde{e}+\tilde{i}+\tilde{r}=1\right)$ and δp<1, it is easy to get that

$$\begin{array}{c} \underset{t\to \infty }{\lim\;\inf}\langle \tilde{i}\left(t\right)\rangle \geq \underline{\tilde{i}}>0\;a.s. \end{array}$$

**Step II.** Similarly, using the Itô’s formula and system (6), we have

(14) $$\begin{array}{cc} d\left(\ln\tilde{e}+\ln\tilde{i}\right)= & [\frac{\left(1-\delta p\right)\beta \tilde{s}\tilde{i}}{\tilde{e}}+\frac{\alpha \tilde{e}}{\tilde{i}}+2\varepsilon \tilde{i}+\sigma_{1}^{2}{\tilde{s}}^{2}+\sigma_{2}^{2}{\tilde{e}}^{2}+\sigma_{3}^{2}{\tilde{i}}^{2}+\sigma_{4}^{2}{\left(1-\tilde{s}-\tilde{e}-\tilde{i}\right)}^{2}\\ & -\left(2b+\alpha +\varepsilon +\gamma +\frac{1}{2}\sigma_{2}^{2}+\frac{1}{2}\sigma_{3}^{2}\right)]dt-2\sigma_{1}\tilde{s}dB_{1}\left(t\right)+\sigma_{2}\left(1-2\tilde{e}\right)dB_{2}\left(t\right)\\ & +\sigma_{3}\left(1-2\tilde{i}\right)dB_{3}\left(t\right)-2\sigma_{4}\left(1-\tilde{s}-\tilde{e}-\tilde{i}\right)dB_{4}\left(t\right). \end{array}$$

Integrating from 0 to *t* and dividing by *t* on both sides of (14) result in

$$\begin{array}{cc} \frac{\ln\tilde{e}\left(t\right)}{t}+\frac{\ln\tilde{i}\left(t\right)}{t}= & \left(1-\delta p\right)\beta \langle \frac{\tilde{s}\tilde{i}}{\tilde{e}}\rangle +\alpha \langle \frac{\tilde{e}}{\tilde{i}}\rangle +2\varepsilon \langle \tilde{i}\rangle +\langle \sigma_{1}^{2}{\tilde{s}}^{2}+\sigma_{2}^{2}{\tilde{e}}^{2}+\sigma_{3}^{2}{\tilde{i}}^{2}+\sigma_{4}^{2}{\left(1-\tilde{s}-\tilde{e}-\tilde{i}\right)}^{2}\rangle \\ & -\left(2b+\alpha +\varepsilon +\gamma +\frac{1}{2}\sigma_{2}^{2}+\frac{1}{2}\sigma_{3}^{2}\right)+\frac{\ln\tilde{e}\left(0\right)}{t}+\frac{\ln\tilde{i}\left(0\right)}{t}+\frac{\hat{M}\left(t\right)}{t}\\ \geq & 2\varepsilon \langle \tilde{i}\rangle -\left(2b+\alpha +\varepsilon +\gamma +\frac{1}{2}\sigma_{2}^{2}+\frac{1}{2}\sigma_{3}^{2}\right)+\frac{\ln\tilde{e}\left(0\right)}{t}+\frac{\ln\tilde{i}\left(0\right)}{t}+\frac{\hat{M}\left(t\right)}{t}, \end{array}$$

here

$$\begin{array}{cc} \hat{M}\left(t\right)= & -2\sigma_{1}\int_{0}^{t}\tilde{s}\left(r\right)dB_{1}\left(r\right)+\sigma_{2}\int_{0}^{t}\left(1-2\tilde{e}\left(r\right)\right)dB_{2}\left(r\right)+\sigma_{3}\int_{0}^{t}\left(1-2\tilde{i}\left(r\right)\right)dB_{3}\left(r\right)\\ & -2\sigma_{4}\int_{0}^{t}\left(1-\tilde{s}\left(r\right)-\tilde{e}\left(r\right)-\tilde{i}\left(r\right)\right)dB_{4}\left(r\right). \end{array}$$

In a similar way as [38], using the strong law of large numbers [37], we have

$$\lim_{t\to \infty }\frac{\hat{M}\left(t\right)}{t}=0\;a.s.$$

Therefore,

$$\begin{array}{c} \underset{t\to \infty }{\lim\;\sup}\langle \tilde{i}\left(t\right)\rangle \leq \bar{\tilde{i}}>0\;a.s. \end{array}$$

The proof of Theorem 3 is complete. ☐

## 5. Stationary Distribution and Ergodicity

Recently, the stationary distribution attract deep research interests of many authors [32,33,34,35]. The ergodicity is one of the most important properties for the stochastic system, and geometric ergodicity for finite-dimensional systems has been shown in detail and well-developed in many earlier works [39,40]. In this section, based on the theory of Khasminskii [41] and the Lyapunov function method, we explore the conditions of the existence of an ergodic stationary distribution, which shows that the epidemic disease will prevail.

Assume X(t) be a time-homogeneous Markov process in $D_{n}\subset R^{n}$, which is described by the stochastic differential equation

$$dX\left(t\right)=b\left(X\right)dt+\sum_{\eta =1}^{n}\sigma_{\eta }\left(X\right)dB_{\eta }\left(t\right),$$

here $D_{n}$ stands for a n-dimensional Euclidean space.

The diffusion matrix is as follows:

$$A\left(x\right)=\left(a_{ij}\left(x\right)\right),\;a_{ij}\left(x\right)=\sum_{\eta =1}^{n}\sigma_{\eta }^{i}\left(x\right)\sigma_{\eta }^{j}\left(x\right).$$

**Assumption** **1.** *Assume that there exists a bounded domain $U\subset D_{n}$ with regular boundary* Γ *such that $\bar{U}\subset D_{n}($$\bar{U}$ is the closure of U), satisfying the following properties:*

(*i*) *In the domain U and some neighborhood thereof, the smallest eigenvalue of the diffusion matrix A(x) is bounded away from zero.*

(*ii*) *If $x\in D_{n}\backslash U$, the mean time τ at which a path issuing from x reaches the set U is finite, and $\underset{x\in \Theta }{\sup}E_{x}\tau <\infty$ for every compact subset $\Theta \subset D_{n}$.*

**Lemma** **3.** *[41] When Assumption 1 holds, then the Markov process X(t) has a stationary distribution π(·). In addition, when f(·) is a function integrable with respect to the measure π, then*

$$\begin{array}{c} P_{x}\left\{\lim_{T\to \infty }\frac{1}{T}\int_{0}^{T}f\left(X\left(t\right)\right)dt=\int_{D_{n}}f\left(x\right)\pi \left(dx\right)\right\}=1 \end{array}$$

*for all $x\in D_{n}$.*

**Remark** **1.** *To prove Assumption 1(i) [42], it suffices to demonstrate that F is uniformly elliptical in any bounded domain H, here*

$$Fu=b\left(x\right)u_{x}+\frac{1}{2}\mathrm{trace}\left(A\left(x\right)u_{xx}\right),$$

*namely, there exists a positive number Z such that*

$$\sum_{i,j=1}^{n}a_{ij}\left(x\right)\xi_{i}\xi_{j}\geq Z{\left|\xi \right|}^{2},\;x\in \bar{H},\;\xi \in R^{n}.$$

*To prove Assumption 1(ii) [43], it suffices to demonstrate that there exist some neighborhood U and a nonnegative $C^{2}$-function V such that $\forall x\in D_{n}\backslash U$, LV(x)<0.*

Making use of the Lemma 3, we can obtain the main results as follows.

**Theorem** **4.** *Let $\left(\tilde{s}\left(t\right),\tilde{e}\left(t\right),\tilde{i}\left(t\right)\right)$ be the solution of system (6) with the initial value $\left(\tilde{s}\left(0\right),\tilde{e}\left(0\right),\tilde{i}\left(0\right)\right)$$\in R_{+}^{3}$ and $\tilde{s}\left(0\right)+\tilde{e}\left(0\right)+\tilde{i}\left(0\right)<1$. If the parameter condition*

$$\sqrt[{3}]{b(1-\delta p)\beta \alpha }>\frac{2\delta p+5b+\alpha +2\varepsilon +2\gamma +2\sigma_{1}^{2}+2\sigma_{2}^{2}+2\sigma_{3}^{2}+\frac{1}{2}\sigma_{4}^{2}}{3}$$

*holds, then the system (6) has a unique stationary distribution π(·) and it has ergodic property.*

**Proof.**  Now let us define a positive-definite function *V* by

$$V=-\ln\left(\tilde{s}+\tilde{e}+\tilde{i}\right)-\ln\tilde{s}-\ln\tilde{e}-\ln\tilde{i}-\ln\tilde{r}.$$

Using the Itô’s formula yields

(15) $$LV\leq -\frac{b}{\tilde{s}}-\frac{\left(1-\delta p\right)\beta \tilde{s}\tilde{i}}{\tilde{e}}-\frac{\alpha \tilde{e}}{\tilde{i}}-\frac{b}{\tilde{s}+\tilde{e}+\tilde{i}}-\frac{\delta p\tilde{s}}{\tilde{r}}-\frac{\gamma \tilde{i}}{\tilde{r}}+M_{1},$$

here

$$M_{1}=\left(1-\delta p\right)\beta +2\delta p+5b+\alpha +2\varepsilon +2\gamma +2\sigma_{1}^{2}+2\sigma_{2}^{2}+2\sigma_{3}^{2}+\frac{1}{2}\sigma_{4}^{2}.$$

The detail derivation process for the above inequality of LV is given in Appendix D.

Next let us construct the following compact subset *U*:

$$U=\left\{\left(\tilde{s},\tilde{e},\tilde{i}\right)\in \tilde{U}:\psi_{1}\leq \tilde{s}<1,\psi_{2}\leq \tilde{e}<1,\psi_{3}\leq \tilde{i}<1,\psi_{4}\leq \tilde{s}+\tilde{e}+\tilde{i}\leq 1-\psi_{4}\right\},$$

where

$$\tilde{U}=\left\{0<\tilde{s}<1,0<\tilde{e}<1,0<\tilde{i}<1,0<\tilde{s}+\tilde{e}+\tilde{i}<1\right\}$$

and $\psi_{i}\in \left(0,1\right)\left(i=1,2,3,4\right)$ is a sufficiently small constant satisfying the following conditions:

(16) $$\psi_{2}={\psi_{1}}^{2}\psi_{3},\;\psi_{4}={\psi_{1}}^{2}={\psi_{3}}^{2},$$

(17) $$-\frac{b}{\psi_{1}}+M_{1}\leq -1,$$

(18) $$-\frac{(1-\delta p)\beta }{\psi_{1}}+M_{1}\leq -1,\;$$

(19) $$\left(1-\delta p\right)\beta \psi_{3}-b<0,$$

(20) $$-\frac{b}{\psi_{4}}+M_{1}\leq -1,$$

(21) $$-\frac{\delta p}{\psi_{1}}-\frac{\gamma }{\psi_{3}}+M_{1}\leq -1.$$

Then

$$\tilde{U}\backslash U=U_{1}\cup U_{2}\cup U_{3}\cup U_{4}\cup U_{5},$$

with

$$U_{1}=\left\{\left(\tilde{s},\tilde{e},\tilde{i}\right)\in \tilde{U}:0<\tilde{s}<\psi_{1}\right\},U_{2}=\left\{\left(\tilde{s},\tilde{e},\tilde{i}\right)\in \tilde{U}:\psi_{1}\leq \tilde{s}<1,0<\tilde{e}<\psi_{2},\psi_{3}\leq \tilde{i}<1\right\},$$

$$U_{3}=\left\{\left(\tilde{s},\tilde{e},\tilde{i}\right)\in \tilde{U}:0<\tilde{i}<\psi_{3}\right\},U_{4}=\left\{\left(\tilde{s},\tilde{e},\tilde{i}\right)\in \tilde{U}:0<\tilde{s}+\tilde{e}+\tilde{i}<\psi_{4}\right\},$$

$$U_{5}=\left\{\left(\tilde{s},\tilde{e},\tilde{i}\right)\in \tilde{U}:\psi_{1}\leq \tilde{s}<1,\psi_{3}\leq \tilde{i}<1,1-\psi_{4}<\tilde{s}+\tilde{e}+\tilde{i}<1\right\}.$$

Now we prove the negativity of LV for any $\tilde{U}\backslash U$.

**Case I.** If $\left(\tilde{s},\tilde{e},\tilde{i}\right)\in U_{1}$, it follows from (A1) and (17) that

$$\begin{array}{c} LV\leq -\frac{b}{\tilde{s}}+M_{1}\leq -\frac{b}{\psi_{1}}+M_{1}\leq -1. \end{array}$$

**Case II.** If $\left(\tilde{s},\tilde{e},\tilde{i}\right)\in U_{2}$, (16) and (18) derive that

$$\begin{array}{c} LV\leq -\frac{\left(1-\delta p\right)\beta \tilde{s}\tilde{i}}{\tilde{e}}+M_{1}\leq -\frac{\left(1-\delta p\right)\beta \psi_{1}\psi_{3}}{\psi_{2}}+M_{1}=-\frac{(1-\delta p)\beta }{\psi_{1}}+M_{1}\leq -1. \end{array}$$

**Case III.** If $\left(\tilde{s},\tilde{e},\tilde{i}\right)\in U_{3}$, (A1) and (19) yield that

$$\begin{array}{cc} LV\leq & -b-\frac{b}{\tilde{s}}-\frac{\left(1-\delta p\right)\beta \tilde{s}\tilde{i}}{\tilde{e}}-\frac{\alpha \tilde{e}}{\tilde{i}}+\left(1-\delta p\right)\beta \psi_{3}+2\delta p+5b+\alpha +2\varepsilon +2\gamma \\ & +2\sigma_{1}^{2}+2\sigma_{2}^{2}+2\sigma_{3}^{2}+\frac{1}{2}\sigma_{4}^{2}\\ \leq & -3\sqrt[{3}]{b(1-\delta p)\beta \alpha }+2\delta p+5b+\alpha +2\varepsilon +2\gamma +2\sigma_{1}^{2}+2\sigma_{2}^{2}+2\sigma_{3}^{2}+\frac{1}{2}\sigma_{4}^{2}<0. \end{array}$$

**Case IV.** If $\left(\tilde{s},\tilde{e},\tilde{i}\right)\in U_{4}$, (A1) and (20) imply that

$$\begin{array}{c} LV\leq -\frac{b}{\tilde{s}+\tilde{e}+\tilde{i}}+M_{1}\leq -\frac{b}{\psi_{4}}+M_{1}\leq -1. \end{array}$$

**Case V.** If $\left(\tilde{s},\tilde{e},\tilde{i}\right)\in U_{5}$, it follows from (A1), (16) and (21) that

$$\begin{array}{c} LV\leq -\frac{\delta p\tilde{s}}{\tilde{r}}-\frac{\gamma \tilde{i}}{\tilde{r}}+M_{1}\leq -\frac{\delta p\psi_{1}}{\psi_{4}}-\frac{\gamma \psi_{3}}{\psi_{4}}+M_{1}=-\frac{\delta p}{\psi_{1}}-\frac{\gamma }{\psi_{3}}+M_{1}\leq -1. \end{array}$$

Define

$$\phi =\max\left\{-1,-3\sqrt[{3}]{b(1-\delta p)\beta \alpha }+2\delta p+5b+\alpha +2\varepsilon +2\gamma +2\sigma_{1}^{2}+2\sigma_{2}^{2}+2\sigma_{3}^{2}+\frac{1}{2}\sigma_{4}^{2}\right\}<0.$$

Obviously, one can see that LV≤ϕ<0 for all $\left(\tilde{s},\tilde{e},\tilde{i}\right)\in \tilde{U}\backslash U$, which shows that Assumption 1(ii) is satisfied. On the other hand, there exists a positive number

$$\begin{array}{cc} Z= & \min\{\left(\sigma_{1}^{2}{\left(1-\tilde{s}\right)}^{2}+\sigma_{2}^{2}{\tilde{e}}^{2}+\sigma_{3}^{2}{\tilde{i}}^{2}+\sigma_{4}^{2}{\left(1-\tilde{s}-\tilde{e}-\tilde{i}\right)}^{2}\right){\tilde{s}}^{2},(\sigma_{1}^{2}{\tilde{s}}^{2}+\sigma_{2}^{2}{\left(1-\tilde{e}\right)}^{2}+\sigma_{3}^{2}{\tilde{i}}^{2}+\sigma_{4}^{2}\\ & \times {\left(1-\tilde{s}-\tilde{e}-\tilde{i}\right)}^{2}){\tilde{e}}^{2},\left(\sigma_{1}^{2}{\tilde{s}}^{2}+\sigma_{2}^{2}{\tilde{e}}^{2}+\sigma_{3}^{2}{\left(1-\tilde{i}\right)}^{2}+\sigma_{4}^{2}{\left(1-\tilde{s}-\tilde{e}-\tilde{i}\right)}^{2}\right){\tilde{i}}^{2},\left(\tilde{s},\tilde{e},\tilde{i}\right)\in \tilde{U}\} \end{array}$$

such that

$$\begin{array}{cc} \sum_{i,j=1}^{3}a_{ij}\xi_{i}\xi_{j}= & \left(\sigma_{1}^{2}{\left(1-\tilde{s}\right)}^{2}+\sigma_{2}^{2}{\tilde{e}}^{2}+\sigma_{3}^{2}{\tilde{i}}^{2}+\sigma_{4}^{2}{\left(1-\tilde{s}-\tilde{e}-\tilde{i}\right)}^{2}\right){\tilde{s}}^{2}\xi_{1}^{2}+(\sigma_{1}^{2}{\tilde{s}}^{2}+\sigma_{2}^{2}{\left(1-\tilde{e}\right)}^{2}+\sigma_{3}^{2}{\tilde{i}}^{2}\\ & +\sigma_{4}^{2}{\left(1-\tilde{s}-\tilde{e}-\tilde{i}\right)}^{2}){\tilde{e}}^{2}\xi_{2}^{2}+\left(\sigma_{1}^{2}{\tilde{s}}^{2}+\sigma_{2}^{2}{\tilde{e}}^{2}+\sigma_{3}^{2}{\left(1-\tilde{i}\right)}^{2}+\sigma_{4}^{2}{\left(1-\tilde{s}-\tilde{e}-\tilde{i}\right)}^{2}\right){\tilde{i}}^{2}\xi_{3}^{2}\\ \geq & Z{\left|\xi \right|}^{2},\;\left(\tilde{s},\tilde{e},\tilde{i}\right)\in \tilde{U},\;\xi \in R^{3}, \end{array}$$

which shows that Assumption 1(i) is satisfied. Consequently, the system (6) has a unique stationary distribution π(·) and it has ergodic property. The proof of Theorem 4 is complete. ☐

## 6. Simulations and Conclusions

### 6.1. Simulations

Next, in order to support the results of the above theorems, we carry out some computer simulations.

**Figure 1:**
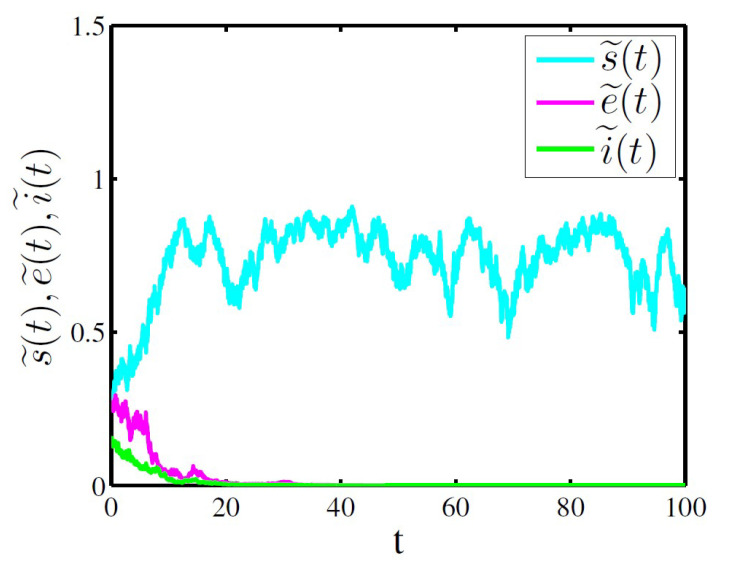
Time sequence diagram of system (6) for extinctions of the exposed and infective individuals.

In Figure 1, take $\tilde{s}\left(0\right)=0.3$, $\tilde{e}\left(0\right)=0.25$, $\tilde{i}\left(0\right)=0.15$, b=0.15, β=0.5, γ=0.3, α=0.2, δ=0.25, p=0.2, ε=0.15 and $\sigma_{1}=\sigma_{2}=\sigma_{3}=\sigma_{4}=0.25$. Then

$$M_{1}=\max\left\{\sigma_{1}^{2},\left(1-\varrho^{2}\right)\sigma_{3}^{2},\sigma_{4}^{2}\right\}=0.0625<2\left(1-\varrho \right)\alpha =0.0652$$

and

ε=0.15<(1−δp)β=0.475<ε+γ+b=0.6

satisfy the parameter conditions in Theorem 2, we can get that the exposed and infective individuals go to extinction almost surely. Obviously, Figure 1 validates our results of the Theorem 2.

**Figure 2:**
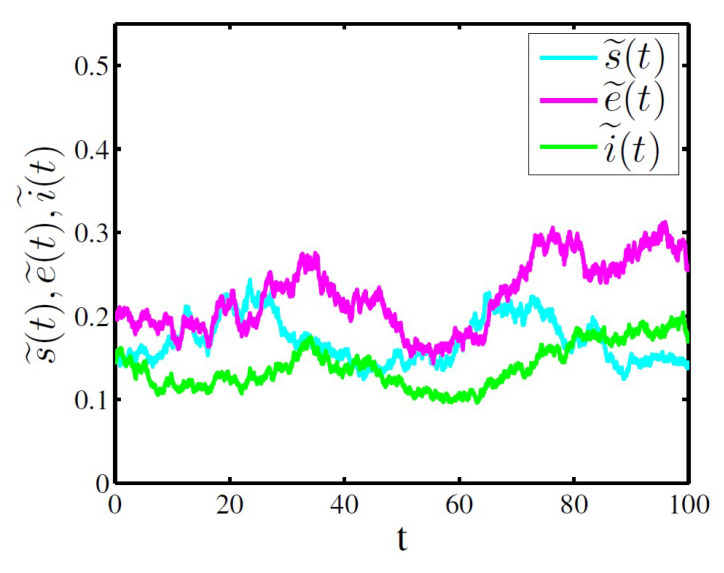
Time sequence diagram of system (6) for permanence in mean of the infective individuals.

In Figure 2, take $\tilde{s}\left(0\right)=0.15$, $\tilde{e}\left(0\right)=0.2$, $\tilde{i}\left(0\right)=0.15$, b=0.02, β=0.9, γ=0.01, α=0.1, δ=0.02, p=0.02, ε=0.16, $\sigma_{1}=0.05$, $\sigma_{2}=0.05$, $\sigma_{3}=0.05$ and $\sigma_{4}=0.1$. Obviously,

$$0.1216=\sqrt[{3}]{b(1-\delta p)\beta \alpha }>\frac{\delta p+3b+\alpha +\varepsilon +\gamma +\frac{1}{2}\sigma_{1}^{2}+\frac{1}{2}\sigma_{2}^{2}+\frac{1}{2}\sigma_{3}^{2}}{3}=0.1114$$

satisfies the parameter condition in Theorem 3, then

$$0.0342=\underline{\tilde{i}}\leq \underset{t\to \infty }{\lim\;\inf}\langle \tilde{i}\left(t\right)\rangle \leq \underset{t\to \infty }{\lim\;\sup}\langle \tilde{i}\left(t\right)\rangle \leq \bar{\tilde{i}}=0.9766,$$

we can get that the infective individuals $\tilde{i}\left(t\right)$ are permanent in mean almost surely. As expected, Figure 2 confirms our results of the Theorem 3.

**Figure 3:**
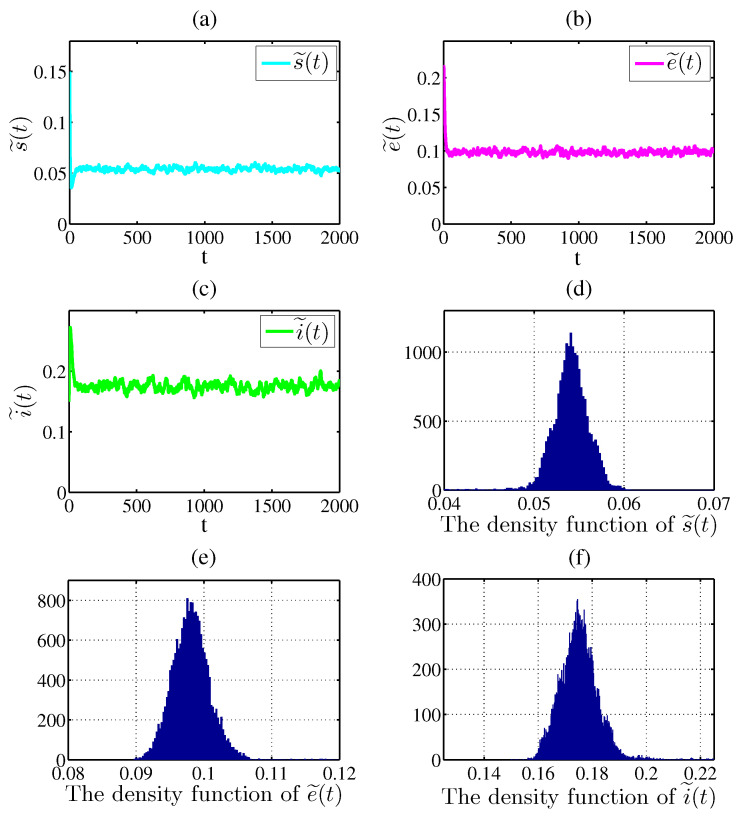
(**a**–**c**) represent the solutions of system (6); (**d**–**e**) stand for the density functions of $\tilde{s}\left(t\right)$, $\tilde{e}\left(t\right)$ and $\tilde{i}\left(t\right)$, respectively.

From Figure 2 and Figure 3, a set of large stochastic parameter values $\sigma_{1}=\sigma_{2}=\sigma_{3}=\sigma_{4}=0.25$ can lead to infective individuals go to extinction (see Figure 2), while infective individuals can be permanent in mean under the condition of a set of small stochastic parameter values $\sigma_{1}=\sigma_{2}=\sigma_{3}=0.05$ and $\sigma_{4}=0.1$ (see Figure 3).

In Figure 3, take $\tilde{s}\left(0\right)=0.15$, $\tilde{e}\left(0\right)=0.2$, $\tilde{i}\left(0\right)=0.15$, b=0.02, β=2.1, γ=0.01, α=0.2, δ=0.02, p=0.02, ε=0.1 and $\sigma_{1}=\sigma_{2}=\sigma_{3}=\sigma_{4}=0.01$. Then

$$0.2033=\sqrt[{3}]{b(1-\delta p)\beta \alpha }>\frac{2\delta p+5b+\alpha +2\varepsilon +2\gamma +2\sigma_{1}^{2}+2\sigma_{2}^{2}+2\sigma_{3}^{2}+\frac{1}{2}\sigma_{4}^{2}}{3}=0.1738$$

satisfies the parameter condition in Theorem 4, we can get that the stochastic system (6) has a unique stationary distribution π(·) and it has ergodic property. Figure 3 indicates that the solution of system (6) swings up and down in a small neighborhood. According to the density functions in Figure 3d–f, we can see that there exists a stationary distribution. As expected, Figure 3 supports our results of the Theorem 4.

The Figure 1, Figure 2 and Figure 3 above show that the large white noise value can lead to infectious diseases to go to extinction, which implies that stochastic fluctuations can suppress the disease outbreak, while the small white noise value can cause infectious diseases to be persistent. In addition, The Figure 3 also shows the stochastic system (6) has a unique ergodic stationary distribution under appropriate conditions. Therefore, the numerical simulation examples are completely consistent with the theoretical results of the Theorems 2–4.

### 6.2. Conclusions

In this paper, we apply stochastic analysis methods to study the global dynamics of a high-dimensional stochastic reduced proportional SEIR epidemic system which makes the analysis novel and complex. We obtain the existence of a unique global positive solution and parameter conditions of extinction or permanence in mean. Furthermore, the solution of the stochastic system has a unique ergodic stationary distribution under certain sufficient parameter conditions. Cubic terms of $\tilde{s},\tilde{e},\tilde{i}$ and multiple stochastic terms for $dB_{i}\left(t\right)\left(i=1,2,3,4\right)$ in system (6) make the analysis more difficult and complex than the models in [34,36]. Some ingenious inequality techniques are used to deal with cubic terms of $\tilde{s},\tilde{e},\tilde{i}$ of system (6). Therefore, compare with previous methods and research results, we develop previous methods and improve the main results of previous studies.

We summarize the main conclusions as follows:

(I) When

$$M_{1}<2\left(1-\varrho \right)\alpha ,\;\varepsilon \leq \left(1-\delta p\right)\beta <\varepsilon +\gamma +b$$

hold, then

$$\underset{t\to \infty }{\lim\;\sup}\frac{\ln\left(\tilde{e}\left(t\right)+\varrho \tilde{i}\left(t\right)\right)}{t}<0\;a.s.$$

That is to say, the exposed and infective individuals go to extinction almost surely.

(II) When

$$\sqrt[{3}]{b(1-\delta p)\beta \alpha }>\frac{\delta p+3b+\alpha +\varepsilon +\gamma +\frac{1}{2}\sigma_{1}^{2}+\frac{1}{2}\sigma_{2}^{2}+\frac{1}{2}\sigma_{3}^{2}}{3}$$

holds, then

$$\underline{\tilde{i}}\leq \underset{t\to \infty }{\lim\;\inf}\langle \tilde{i}\left(t\right)\rangle \leq \underset{t\to \infty }{\lim\;\sup}\langle \tilde{i}\left(t\right)\rangle \leq \bar{\tilde{i}}\;a.s.$$

That is to say, the infective individuals $\tilde{i}\left(t\right)$ are permanent in mean almost surely.

(III) When

$$\sqrt[{3}]{b(1-\delta p)\beta \alpha }>\frac{2\delta p+5b+\alpha +2\varepsilon +2\gamma +2\sigma_{1}^{2}+2\sigma_{2}^{2}+2\sigma_{3}^{2}+\frac{1}{2}\sigma_{4}^{2}}{3}$$

holds, then the system (6) has a unique stationary distribution π(·) and it has ergodic property.

By comparing the above conclusions (II) and (III), we can see that when system (6) has a ergodic stationary distribution, then the infective individuals $\tilde{i}\left(t\right)$ are permanent in mean almost surely. However, it is not applicable in reverse. The above results of Theorems 2–4 show a large stochastic disturbance can cause infectious diseases to go to extinction, in other words, the persistent infectious disease of a deterministic system can become extinct due to the white noise stochastic disturbance. This implies that stochastic fluctuations can suppress the disease outbreak.

## Appendix A.

**Proof** **of** **Theorem** **1.** Define a $C^{2}$-function *V*: $R_{+}^{3}\to R_{+}$ by

$$\begin{array}{cc} V\left(\tilde{s},\tilde{e},\tilde{i}\right)= & -\ln\left(1-\tilde{s}-\tilde{e}-\tilde{i}\right)-\ln\tilde{s}-\ln\tilde{e}-\ln\tilde{i}-3. \end{array}$$

$$\begin{array}{cc} dV= & LVdt+\sigma_{1}\left(4\tilde{s}-1\right)dB_{1}\left(t\right)+\sigma_{2}\left(4\tilde{e}-1\right)dB_{2}\left(t\right)+\sigma_{3}\left(4\tilde{i}-1\right)dB_{3}\left(t\right)\\ & +\sigma_{4}\left(3-4\tilde{s}-4\tilde{e}-4\tilde{i}\right)dB_{4}\left(t\right), \end{array}$$

where

$$\begin{array}{cc} LV= & \frac{b\left(2\tilde{s}+\tilde{e}+\tilde{i}-1\right)}{\tilde{s}\left(1-\tilde{s}-\tilde{e}-\tilde{i}\right)}-\frac{\left(1-\delta p\right)\beta \tilde{i}\left(2\tilde{s}+\tilde{e}+\tilde{i}-1\right)}{1-\tilde{s}-\tilde{e}-\tilde{i}}-\frac{\left(\delta p+b\right)\left(2\tilde{s}+\tilde{e}+\tilde{i}-1\right)}{1-\tilde{s}-\tilde{e}-\tilde{i}}\\ & +\frac{\varepsilon \tilde{i}\left(4\tilde{s}+4\tilde{e}+4\tilde{i}-3\right)}{1-\tilde{s}-\tilde{e}-\tilde{i}}+\frac{\left(\sigma_{1}^{2}{\tilde{s}}^{2}+\sigma_{2}^{2}{\tilde{e}}^{2}+\sigma_{3}^{2}{\tilde{i}}^{2}+\sigma_{4}^{2}{\left(1-\tilde{s}-\tilde{e}-\tilde{i}\right)}^{2}\right)\left(4\tilde{s}+4\tilde{e}+4\tilde{i}-3\right)}{1-\tilde{s}-\tilde{e}-\tilde{i}}\\ & -\frac{\sigma_{1}^{2}\tilde{s}\left(2\tilde{s}+\tilde{e}+\tilde{i}-1\right)}{1-\tilde{s}-\tilde{e}-\tilde{i}}+\frac{\left(1-\delta p\right)\beta \tilde{s}\tilde{i}\left(\tilde{s}+2\tilde{e}+\tilde{i}-1\right)}{\tilde{e}\left(1-\tilde{s}-\tilde{e}-\tilde{i}\right)}-\frac{\left(\alpha +b\right)\left(\tilde{s}+2\tilde{e}+\tilde{i}-1\right)}{1-\tilde{s}-\tilde{e}-\tilde{i}}\\ & -\frac{\sigma_{2}^{2}\tilde{e}\left(\tilde{s}+2\tilde{e}+\tilde{i}-1\right)}{1-\tilde{s}-\tilde{e}-\tilde{i}}+\frac{\alpha \tilde{e}\left(\tilde{s}+\tilde{e}+2\tilde{i}-1\right)}{\tilde{i}\left(1-\tilde{s}-\tilde{e}-\tilde{i}\right)}-\frac{\left(\varepsilon +\gamma +b\right)\left(\tilde{s}+\tilde{e}+2\tilde{i}-1\right)}{1-\tilde{s}-\tilde{e}-\tilde{i}}\\ & -\frac{\sigma_{3}^{2}\tilde{i}\left(\tilde{s}+\tilde{e}+2\tilde{i}-1\right)}{1-\tilde{s}-\tilde{e}-\tilde{i}}+\frac{1}{2}\sigma_{1}^{2}\left(4{\tilde{s}}^{2}-2\tilde{s}+1\right)+\frac{1}{2}\sigma_{2}^{2}\left(4{\tilde{e}}^{2}-2\tilde{e}+1\right)+\frac{1}{2}\sigma_{3}^{2}\left(4{\tilde{i}}^{2}-2\tilde{i}+1\right)\\ & +\frac{1}{2}\sigma_{4}^{2}\left[3{\left(1-\tilde{s}-\tilde{e}-\tilde{i}\right)}^{2}+{\left(\tilde{s}+\tilde{e}+\tilde{i}\right)}^{2}\right], \end{array}$$

since $\tilde{r}=1-\tilde{s}-\tilde{e}-\tilde{i}$ and δp<1, thus

$$\begin{array}{cc} LV\leq & \frac{b\left(\tilde{s}-\tilde{r}\right)}{\tilde{s}\tilde{r}}-\frac{\left(1-\delta p\right)\beta \tilde{i}\left(\tilde{s}-\tilde{r}\right)}{\tilde{r}}-\frac{\left(\delta p+b\right)\left(\tilde{s}-\tilde{r}\right)}{\tilde{r}}+\frac{\varepsilon \tilde{i}\left(1-4\tilde{r}\right)}{\tilde{r}}+\frac{\left(1-\delta p\right)\beta \tilde{s}\tilde{i}\left(\tilde{e}-\tilde{r}\right)}{\tilde{e}\tilde{r}}\\ & -\frac{\left(\alpha +b\right)\left(\tilde{e}-\tilde{r}\right)}{\tilde{r}}+\frac{\alpha \tilde{e}\left(\tilde{i}-\tilde{r}\right)}{\tilde{i}\tilde{r}}-\frac{\left(\varepsilon +\gamma +b\right)\left(\tilde{i}-\tilde{r}\right)}{\tilde{r}}+\frac{1}{2}\sigma_{1}^{2}+\frac{1}{2}\sigma_{2}^{2}+\frac{1}{2}\sigma_{3}^{2}+\frac{1}{2}\sigma_{4}^{2}\\ = & \frac{b}{\tilde{r}}-\frac{b}{\tilde{s}}-\frac{\beta \tilde{s}\tilde{i}}{\tilde{r}}+\beta \tilde{i}+\frac{\beta \delta p\tilde{s}\tilde{i}}{\tilde{r}}-\beta \delta p\tilde{i}-\frac{\delta p\tilde{s}}{\tilde{r}}+\delta p-\frac{b\tilde{s}}{\tilde{r}}+b+\frac{\varepsilon \tilde{i}}{\tilde{r}}-4\varepsilon \tilde{i}-\frac{\varepsilon \tilde{i}}{\tilde{r}}+\varepsilon +\frac{\beta \tilde{s}\tilde{i}}{\tilde{r}}\\ & -\frac{\beta \tilde{s}\tilde{i}}{\tilde{e}}-\frac{\beta \delta p\tilde{s}\tilde{i}}{\tilde{r}}+\frac{\beta \delta p\tilde{s}\tilde{i}}{\tilde{e}}-\frac{\alpha \tilde{e}}{\tilde{r}}+\alpha -\frac{b\tilde{e}}{\tilde{r}}+b+\frac{\alpha \tilde{e}}{\tilde{r}}-\frac{\alpha \tilde{e}}{\tilde{i}}-\frac{\gamma \tilde{i}}{\tilde{r}}+\gamma -\frac{b\tilde{i}}{\tilde{r}}+b\\ & +\frac{1}{2}\sigma_{1}^{2}+\frac{1}{2}\sigma_{2}^{2}+\frac{1}{2}\sigma_{3}^{2}+\frac{1}{2}\sigma_{4}^{2}\\ \leq & \frac{b}{\tilde{r}}-\frac{b\tilde{s}}{\tilde{r}}-\frac{b\tilde{e}}{\tilde{r}}-\frac{b\tilde{i}}{\tilde{r}}+\beta \tilde{i}+\delta p+3b+\varepsilon +\frac{\beta \tilde{s}\tilde{i}}{\tilde{e}}\left(\delta p-1\right)+\alpha +\gamma +\frac{1}{2}\sigma_{1}^{2}+\frac{1}{2}\sigma_{2}^{2}+\frac{1}{2}\sigma_{3}^{2}+\frac{1}{2}\sigma_{4}^{2}\\ \leq & \beta +\delta p+4b+\varepsilon +\alpha +\gamma +\frac{1}{2}\sigma_{1}^{2}+\frac{1}{2}\sigma_{2}^{2}+\frac{1}{2}\sigma_{3}^{2}+\frac{1}{2}\sigma_{4}^{2}\\ := & M_{0}, \end{array}$$

where $M_{0}$ is a positive constant.  ☐

## Appendix B.

**Proof** **of** **Theorem** **2.**

$$\begin{array}{cc} LV= & \frac{1}{\tilde{e}+\varrho \tilde{i}}\left[\left(1-\delta p\right)\beta \tilde{s}\tilde{i}-\left(\alpha +b\right)\tilde{e}+\varepsilon \tilde{e}\tilde{i}+\varrho \alpha \tilde{e}-\varrho \left(\varepsilon +\gamma +b\right)\tilde{i}+\varrho \varepsilon {\tilde{i}}^{2}\right]+\frac{1}{2}\sigma_{1}^{2}{\tilde{s}}^{2}\\ & +\frac{1}{2}\left[1-\frac{1}{{\left(\tilde{e}+\varrho \tilde{i}\right)}^{2}}\right]\sigma_{2}^{2}{\tilde{e}}^{2}+\frac{1}{2}\left[1-\frac{\varrho^{2}}{{\left(\tilde{e}+\varrho \tilde{i}\right)}^{2}}\right]\sigma_{3}^{2}{\tilde{i}}^{2}+\frac{1}{2}\sigma_{4}^{2}{\left(1-\tilde{s}-\tilde{e}-\tilde{i}\right)}^{2}, \end{array}$$

since $\tilde{s}=1-\tilde{e}-\tilde{i}-\tilde{r}$ and δp<1, thus

$$\begin{array}{cc} LV\leq & \frac{1}{\tilde{e}+\varrho \tilde{i}}[\left(\beta -\delta p\beta -\varrho \left(\varepsilon +\gamma +b\right)\right)\tilde{i}+\left(\varepsilon -\beta +\delta p\beta \right)\tilde{e}\tilde{i}+\left(\varrho \varepsilon -\beta +\delta p\beta \right){\tilde{i}}^{2}\\ & +\left(\varrho -1\right)\alpha \tilde{e}]+\frac{1}{2}\sigma_{1}^{2}{\tilde{s}}^{2}+\frac{1}{2}\left[1-\frac{1}{{\left(\tilde{e}+\varrho \tilde{i}\right)}^{2}}\right]\sigma_{2}^{2}{\tilde{e}}^{2}+\frac{1}{2}\left[1-\frac{\varrho^{2}}{{\left(\tilde{e}+\varrho \tilde{i}\right)}^{2}}\right]\sigma_{3}^{2}{\tilde{i}}^{2}\\ & +\frac{1}{2}\sigma_{4}^{2}{\left(1-\tilde{s}-\tilde{e}-\tilde{i}\right)}^{2}. \end{array}$$

Take

$$\varrho =\frac{-\left(\varepsilon +\gamma +b-\alpha \right)+\sqrt{{\left(\varepsilon +\gamma +b-\alpha \right)}^{2}+4\left(1-\delta p\right)\alpha \beta }}{2\alpha }$$

such that ϱ(ϱ−1)α=β−δpβ−ϱ(ε+γ+b). Here, it is easy to see that ϱ∈(0,1). Then one can derive that

$$\begin{array}{cc} dV\leq & \left[\left(\varrho -1\right)\alpha +\frac{1}{2}\sigma_{1}^{2}{\tilde{s}}^{2}+\frac{1}{2}\left(1-\varrho^{2}\right)\sigma_{3}^{2}{\tilde{i}}^{2}+\frac{1}{2}\sigma_{4}^{2}{\left(1-\tilde{s}-\tilde{e}-\tilde{i}\right)}^{2}\right]dt-\sigma_{1}\tilde{s}dB_{1}\left(t\right)\\ & +\frac{\sigma_{2}\tilde{e}\left(1-\tilde{e}-\varrho \tilde{i}\right)}{\tilde{e}+\varrho \tilde{i}}dB_{2}\left(t\right)+\frac{\sigma_{3}\tilde{i}\left(\varrho -\tilde{e}-\varrho \tilde{i}\right)}{\tilde{e}+\varrho \tilde{i}}dB_{3}\left(t\right)-\sigma_{4}\left(1-\tilde{s}-\tilde{e}-\tilde{i}\right)dB_{4}\left(t\right)\\ \leq & \left[\left(\varrho -1\right)\alpha +\frac{M_{1}}{2}\right]dt-\sigma_{1}\tilde{s}dB_{1}\left(t\right)+\frac{\sigma_{2}\tilde{e}\left(1-\tilde{e}-\varrho \tilde{i}\right)}{\tilde{e}+\varrho \tilde{i}}dB_{2}\left(t\right)+\frac{\sigma_{3}\tilde{i}\left(\varrho -\tilde{e}-\varrho \tilde{i}\right)}{\tilde{e}+\varrho \tilde{i}}dB_{3}\left(t\right)\\ & -\sigma_{4}\left(1-\tilde{s}-\tilde{e}-\tilde{i}\right)dB_{4}\left(t\right), \end{array}$$

here $M_{1}=\max\left\{\sigma_{1}^{2},\left(1-\varrho^{2}\right)\sigma_{3}^{2},\sigma_{4}^{2}\right\}$.  ☐

## Appendix C.

**Proof** **of** **Theorem** **3.**

$$\begin{array}{cc} \frac{\ln\tilde{s}\left(t\right)}{t}+\frac{\ln\tilde{e}\left(t\right)}{t}+\frac{\ln\tilde{i}\left(t\right)}{t}= & b\langle \frac{1}{\tilde{s}}\rangle -\left(1-\delta p\right)\beta \langle \tilde{i}\rangle +3\varepsilon \langle \tilde{i}\rangle +\left(1-\delta p\right)\beta \langle \frac{\tilde{s}\tilde{i}}{\tilde{e}}\rangle +\alpha \langle \frac{\tilde{e}}{\tilde{i}}\rangle \\ & +\langle \frac{3}{2}\sigma_{1}^{2}{\tilde{s}}^{2}+\frac{3}{2}\sigma_{2}^{2}{\tilde{e}}^{2}+\frac{3}{2}\sigma_{3}^{2}{\tilde{i}}^{2}+\frac{3}{2}\sigma_{4}^{2}{\left(1-\tilde{s}-\tilde{e}-\tilde{i}\right)}^{2}\rangle \\ & -\left(\delta p+3b+\alpha +\varepsilon +\gamma +\frac{1}{2}\sigma_{1}^{2}+\frac{1}{2}\sigma_{2}^{2}+\frac{1}{2}\sigma_{3}^{2}\right)+\frac{\ln\tilde{s}\left(0\right)}{t}\\ & +\frac{\ln\tilde{e}\left(0\right)}{t}+\frac{\ln\tilde{i}\left(0\right)}{t}+\frac{\underline{M}\left(t\right)}{t}\\ \geq & \langle b\frac{1}{\tilde{s}}+\left(1-\delta p\right)\beta \frac{\tilde{s}\tilde{i}}{\tilde{e}}+\alpha \frac{\tilde{e}}{\tilde{i}}\rangle -\left(1-\delta p\right)\beta \langle \tilde{i}\rangle -(\delta p+3b+\alpha +\varepsilon \\ & +\gamma +\frac{1}{2}\sigma_{1}^{2}+\frac{1}{2}\sigma_{2}^{2}+\frac{1}{2}\sigma_{3}^{2})+\frac{\ln\tilde{s}\left(0\right)}{t}+\frac{\ln\tilde{e}\left(0\right)}{t}+\frac{\ln\tilde{i}\left(0\right)}{t}+\frac{\underline{M}\left(t\right)}{t}\\ \geq & 3\sqrt[{3}]{b(1-\delta p)\beta \alpha }-\left(1-\delta p\right)\beta \langle \tilde{i}\rangle -(\delta p+3b+\alpha +\varepsilon +\gamma \\ & +\frac{1}{2}\sigma_{1}^{2}+\frac{1}{2}\sigma_{2}^{2}+\frac{1}{2}\sigma_{3}^{2})+\frac{\ln\tilde{s}\left(0\right)}{t}+\frac{\ln\tilde{e}\left(0\right)}{t}+\frac{\ln\tilde{i}\left(0\right)}{t}+\frac{\underline{M}\left(t\right)}{t}. \end{array}$$

 ☐

## Appendix D.

**Proof** **of** **Theorem** **4.** Now let us define a positive-definite function *V* by

$$\begin{array}{cc} V= & -\ln\left(\tilde{s}+\tilde{e}+\tilde{i}\right)-\ln\tilde{s}-\ln\tilde{e}-\ln\tilde{i}-\ln\tilde{r}\\ & :=V_{1}+V_{2}+V_{3}+V_{4}+V_{5}. \end{array}$$

From the Itô’s formula yields

$$\begin{array}{cc} LV_{1}= & -\frac{1}{\tilde{s}+\tilde{e}+\tilde{i}}[b-\left(\delta p+b\right)\tilde{s}+\varepsilon \tilde{s}\tilde{i}-b\tilde{e}+\varepsilon \tilde{e}\tilde{i}-\left(\varepsilon +\gamma +b\right)\tilde{i}+\varepsilon {\tilde{i}}^{2}-\sigma_{1}^{2}{\tilde{s}}^{2}-\sigma_{2}^{2}{\tilde{e}}^{2}-\sigma_{3}^{2}{\tilde{i}}^{2}\\ & +\left(\tilde{s}+\tilde{e}+\tilde{i}\right)\left(\sigma_{1}^{2}{\tilde{s}}^{2}+\sigma_{2}^{2}{\tilde{e}}^{2}+\sigma_{3}^{2}{\tilde{i}}^{2}+\sigma_{4}^{2}{\tilde{r}}^{2}\right)]+\frac{{\tilde{r}}^{2}\left[\sigma_{1}^{2}{\tilde{s}}^{2}+\sigma_{2}^{2}{\tilde{e}}^{2}+\sigma_{3}^{2}{\tilde{i}}^{2}+\sigma_{4}^{2}{\left(\tilde{s}+\tilde{e}+\tilde{i}\right)}^{2}\right]}{2{\left(\tilde{s}+\tilde{e}+\tilde{i}\right)}^{2}}\\ = & -\frac{b}{\tilde{s}+\tilde{e}+\tilde{i}}+\frac{\delta p\tilde{s}+\left(\varepsilon +\gamma \right)\tilde{i}}{\tilde{s}+\tilde{e}+\tilde{i}}-\varepsilon \tilde{i}+\frac{\sigma_{1}^{2}{\tilde{s}}^{2}+\sigma_{2}^{2}{\tilde{e}}^{2}+\sigma_{3}^{2}{\tilde{i}}^{2}}{\tilde{s}+\tilde{e}+\tilde{i}}-\left(\sigma_{1}^{2}{\tilde{s}}^{2}+\sigma_{2}^{2}{\tilde{e}}^{2}+\sigma_{3}^{2}{\tilde{i}}^{2}+\frac{1}{2}\sigma_{4}^{2}{\tilde{r}}^{2}\right)\\ & +\frac{{\tilde{r}}^{2}\left(\sigma_{1}^{2}{\tilde{s}}^{2}+\sigma_{2}^{2}{\tilde{e}}^{2}+\sigma_{3}^{2}{\tilde{i}}^{2}\right)}{2{\left(\tilde{s}+\tilde{e}+\tilde{i}\right)}^{2}}+b. \end{array}$$

Similarly,

$$\begin{array}{cc} LV_{2}= & -\frac{b}{\tilde{s}}+\left(1-\delta p\right)\beta \tilde{i}-\varepsilon \tilde{i}+\delta p+b+\frac{1}{2}\sigma_{1}^{2}-\frac{1}{2}\left(\sigma_{1}^{2}{\tilde{s}}^{2}+\sigma_{2}^{2}{\tilde{e}}^{2}+\sigma_{3}^{2}{\tilde{i}}^{2}+\sigma_{4}^{2}{\tilde{r}}^{2}\right),\\ LV_{3}= & -\frac{\left(1-\delta p\right)\beta \tilde{s}\tilde{i}}{\tilde{e}}-\varepsilon \tilde{i}+\alpha +b+\frac{1}{2}\sigma_{2}^{2}-\frac{1}{2}\left(\sigma_{1}^{2}{\tilde{s}}^{2}+\sigma_{2}^{2}{\tilde{e}}^{2}+\sigma_{3}^{2}{\tilde{i}}^{2}+\sigma_{4}^{2}{\tilde{r}}^{2}\right),\\ LV_{4}= & -\frac{\alpha \tilde{e}}{\tilde{i}}-\varepsilon \tilde{i}+\varepsilon +\gamma +b+\frac{1}{2}\sigma_{3}^{2}-\frac{1}{2}\left(\sigma_{1}^{2}{\tilde{s}}^{2}+\sigma_{2}^{2}{\tilde{e}}^{2}+\sigma_{3}^{2}{\tilde{i}}^{2}+\sigma_{4}^{2}{\tilde{r}}^{2}\right),\\ LV_{5}= & -\frac{\delta p\tilde{s}}{\tilde{r}}-\frac{\gamma \tilde{i}}{\tilde{r}}-\varepsilon \tilde{i}+b+\frac{1}{2}\sigma_{4}^{2}-\frac{1}{2}\left(\sigma_{1}^{2}{\tilde{s}}^{2}+\sigma_{2}^{2}{\tilde{e}}^{2}+\sigma_{3}^{2}{\tilde{i}}^{2}+\sigma_{4}^{2}{\tilde{r}}^{2}\right). \end{array}$$

Therefore,

(A1) $$\begin{array}{cc} LV= & -\frac{b}{\tilde{s}+\tilde{e}+\tilde{i}}-\frac{b}{\tilde{s}}-\frac{\left(1-\delta p\right)\beta \tilde{s}\tilde{i}}{\tilde{e}}-\frac{\alpha \tilde{e}}{\tilde{i}}-5\varepsilon \tilde{i}-\frac{\delta p\tilde{s}}{\tilde{r}}-\frac{\gamma \tilde{i}}{\tilde{r}}+\frac{\delta p\tilde{s}+\left(\varepsilon +\gamma \right)\tilde{i}}{\tilde{s}+\tilde{e}+\tilde{i}}\\ & +\frac{\sigma_{1}^{2}{\tilde{s}}^{2}+\sigma_{2}^{2}{\tilde{e}}^{2}+\sigma_{3}^{2}{\tilde{i}}^{2}}{\tilde{s}+\tilde{e}+\tilde{i}}+\frac{{\tilde{r}}^{2}\left(\sigma_{1}^{2}{\tilde{s}}^{2}+\sigma_{2}^{2}{\tilde{e}}^{2}+\sigma_{3}^{2}{\tilde{i}}^{2}\right)}{2{\left(\tilde{s}+\tilde{e}+\tilde{i}\right)}^{2}}+\left(1-\delta p\right)\beta \tilde{i}-(3\sigma_{1}^{2}{\tilde{s}}^{2}\\ & +3\sigma_{2}^{2}{\tilde{e}}^{2}+3\sigma_{3}^{2}{\tilde{i}}^{2}+\frac{5}{2}\sigma_{4}^{2}{\tilde{r}}^{2})+\frac{1}{2}\left(\sigma_{1}^{2}+\sigma_{2}^{2}+\sigma_{3}^{2}+\sigma_{4}^{2}\right)+\delta p+5b+\alpha +\varepsilon +\gamma \\ \leq & -\frac{b}{\tilde{s}}-\frac{\left(1-\delta p\right)\beta \tilde{s}\tilde{i}}{\tilde{e}}-\frac{\alpha \tilde{e}}{\tilde{i}}-\frac{b}{\tilde{s}+\tilde{e}+\tilde{i}}-\frac{\delta p\tilde{s}}{\tilde{r}}-\frac{\gamma \tilde{i}}{\tilde{r}}+M_{1}, \end{array}$$

here

$$\begin{array}{c} M_{1}=\left(1-\delta p\right)\beta +2\delta p+5b+\alpha +2\varepsilon +2\gamma +2\sigma_{1}^{2}+2\sigma_{2}^{2}+2\sigma_{3}^{2}+\frac{1}{2}\sigma_{4}^{2}. \end{array}$$

 ☐
